# Development of Wireless Power-Transmission-Based Photodynamic Therapy for the Induction of Cell Death in Cancer Cells by Cyclometalated Iridium(III) Complexes

**DOI:** 10.3390/molecules28031433

**Published:** 2023-02-02

**Authors:** Kenta Yokoi, Yoshitaka Yasuda, Azusa Kanbe, Takehiro Imura, Shin Aoki

**Affiliations:** 1 Faculty of Pharmaceutical Sciences, Tokyo University of Science, 2641 Yamazaki, Noda 278-8510, Chiba, Japan; 2 Faculty of Science and Technology, Tokyo University of Science, 2641 Yamazaki, Noda 278-8510, Chiba, Japan; 3 Research Institute for Science and Technology (RIST), Tokyo University of Science, 2641 Yamazaki, Noda 278-8510, Chiba, Japan; 4 Research Institute for Biomedical Sciences (RIBS), Tokyo University of Science, 2641 Yamazaki, Noda 278-8510, Chiba, Japan

**Keywords:** photodynamic therapy (PDT), wireless power transmission (WPT), cyclometalated iridium(III) complex, photosensitizer, singlet oxygen, cancer therapy

## Abstract

Photodynamic therapy (PDT), a noninvasive method for cancer therapy, involves the generation of reactive oxygen species (ROS) by the photochemical excitation of photosensitizers (PSs) to induce cell death in cancer cells. A variety of PS including porphyrin derivatives and metal complexes such as iridium (Ir) complexes have been reported. In clinical trials, red-near infrared (NIR) light (650–900 nm) is preferred for the excitation of PSs due to its deeper penetration into tissues compared with visible light (400–500 nm). To overcome this limitation, we established a PDT system that uses cyclometalated iridium(III) (Ir(III)) complexes that are excited with blue light in the wireless power transmission (WPT) system. To achieve this, we developed a light-emitting diode (LED) light device equipped with a receiver coil that receives electricity from the transmitter coil through magnetic resonance coupling. The LEDs in the receiving device use blue light (470 nm) to irradiate a given Ir(III) complex and excite triplet oxygen (^3^O_2_) to singlet oxygen (^1^O_2_) which induces cell death in HeLa S3 cells (human cervical carcinoma cells). The results obtained in this study suggest that WPT-based PDT represents a potentially new method for the treatment of tumors by a non-battery LED, which are otherwise difficult to treat by previous PDT systems.

## 1. Introduction

**Scheme 1:**
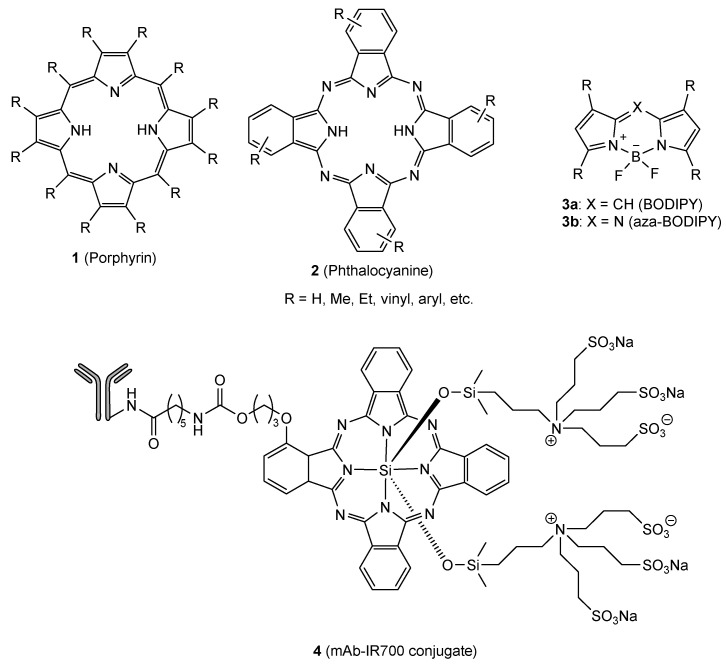
The structures of representative photosensitizers and antibody-silicon-phthalocyanine conjugates based on porphyrin, phthalocyanine, and BODIPY (4,4-difluoro-4-bora-3a,4a-diaza-s-indacene) scaffolds.

Photodynamic therapy (PDT), one of the modalities for noninvasive cancer therapy, has fewer side effects compared with surgery, chemotherapy, and radiotherapy [1,2,3,4,5]. PDT requires light with an appropriate wavelength which excites photoreactive compounds referred to as photosensitizers (PSs) in order to generate reactive oxygen species (ROS), such as singlet oxygen (^1^O_2_) from triplet oxygen (^3^O_2_), for inducing damage to biomolecules such as DNA, proteins, organelles, and cell membrane, thus resulting in cancer cell death through apoptosis and/or necrosis. Therefore, PSs are designed to selectively accumulate in cancer cells and be less cytotoxic without photoirradiation. Numerous examples of PSs, including porphyrin derivatives (**1**), phthalocyanine derivatives (**2**), BODIPY (4,4-difluoro-4-bora-3a,4a-diaza-s-indacene) or aza-BODIPY derivatives (**3**), and metal complexes, have been reported [6,7,8,9,10,11]. Near-infrared photoimmunotherapy (NIR-PIT) of cancer using antibody-silicon-phthalocyanine conjugates such as **4** (monoclonal antibody-IR700 conjugate) have also been reported (Scheme 1) [12,13].

**Scheme 2:**
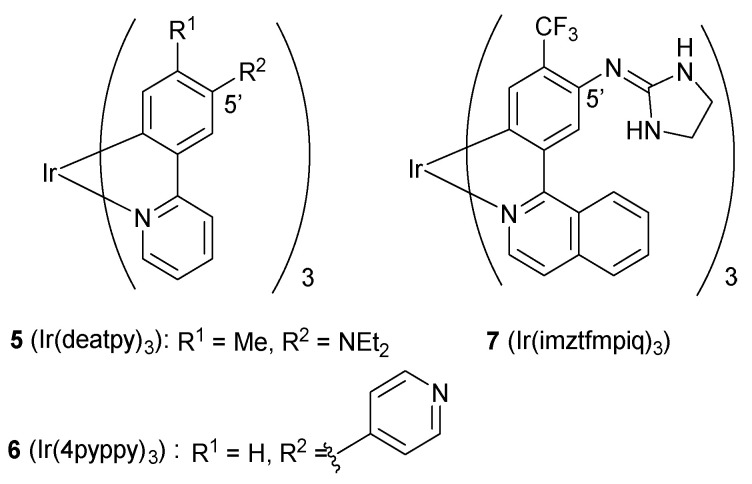
The structures of representative cyclometalated Ir(III) complexes **5**–**7** (deatpy: 2-(5′-N,N-diethylamino-4′-tolyl)pyridine, 4pyppy: 2-(5′-(4′-pyridyl)phenyl)pyridine, imztfmpiq: 1-(5′-iminoimidazolidyl-4′-trifluoromethylphenyl)isoquinoline)).

It has been proposed that cyclometalated iridium(III) (Ir(III)) complexes would be potent candidates of PSs for PDT [14,15,16,17,18,19,20,21,22,23]. We previously reported on the design and synthesis of cyclometalated iridium(III) complexes as luminescence pH sensors and specific probes for intracellular organelles such as mitochondria and lysosomes. These complexes contain cyclometalating ligands such as 2-(4′-tolyl)pyridine (tpy), 2-phenylpyridine (ppy), and 1-(4′-trifluoromethylphenyl)isoquinoline (tfmpiq) modified with basic amino units, including N,N-diethylamino (**5**), 4-pyridyl (**6**), and iminoimidazolidyl (**7**) groups (Scheme 2) [24,25,26,27]. These Ir complexes also function as PSs when they are photoirradiated at 377 nm or 465 nm to produce ^1^O_2_ from ^3^O_2_ and induce necrosis-like cell death in HeLa S3 cells (human cervical carcinoma cells).

**Figure 1:**
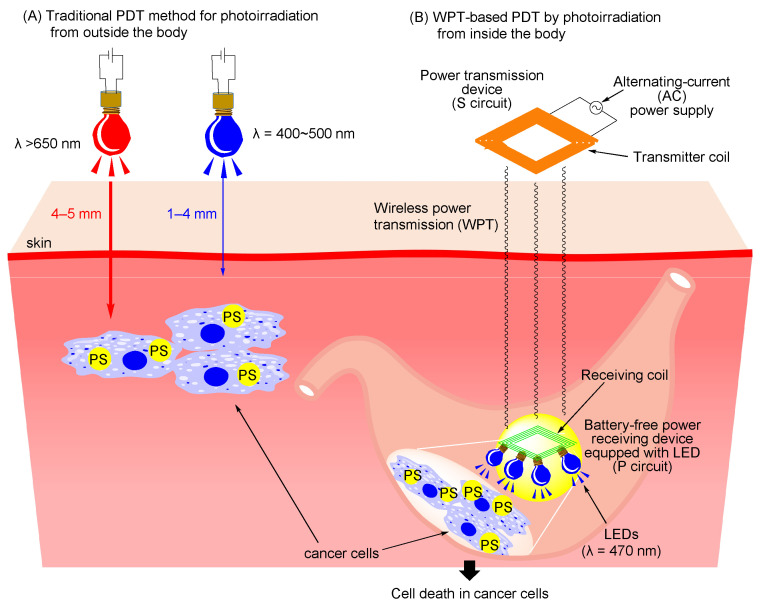
A schematic proposal of wireless power-transmission-based photodynamic therapy of cancer (WPT-PDT), in which photoirradiation is conducted inside the body (**B**), compared with the currently accepted PDT that uses irradiation from outside the body (**A**). PS depicts a photosensitizer.

It is generally considered that red (>650 nm) and near-infrared light (NIR) (700–900 nm) are preferable for the excitation of porphyrin and phthalocyanine derivatives in PDT due to the fact that they penetrate deeper into tissues (4–5 mm), compared with visible light (400–600 nm) that penetrates only to a depth of 1–4 mm (Figure 1A) [28,29]. However, the irradiation of **1** and **2** and their analogs with longer wavelength (>600 nm) light generates ^1^O_2_ in low yields because of their small molar extinction coefficients (*ε*) in the red-NIR regions. As a result, a longer irradiation time is required for the treatment of a tumor. In this context, the recently developed BODIPY-based PSs have higher *ε* values in the NIR region and a higher PDT efficiency by NIR irradiation [30]. On the other hand, the use of shorter wavelength light may allow additional types of PSs to be used in PDT applications.

To overcome these limitations of PSs and the irradiation light, wireless power transmission (WPT) has gained considerable interest for possible use in transferring electric energy from a transmitter coil (transmitting device) to a receiver coil (receiving device) without a direct connection [31,32,33]. In WPT, a transmitting device driven by electric power transfers electric energy from a transmitting device to a receiving device through microwaves, ultrasonic waves, magnetic coupling, and electronic coupling without a direct electrical cable [34,35,36,37,38,39], and this technology has been applied to electric vehicles (EVs) [40,41,42,43], the Internet of Things (IoT) [44,45], and medicinal implants [46,47], as well as related fields. Applications of WPT to PDT have also been reported, in which receiving devices containing light-emitting diodes (LEDs) are implanted onto tumors in living animals, and power is wirelessly transferred from the transmitting device through magnetic coupling to photoirradiate (green or red light) PSs such as photofrin and chlorin e6; this approach has been reported to cause a decrease in tumor volume [48,49,50,51].

We report herein on the development of a WPT-based PDT system that uses shorter wavelength (blue light) to expand the combinations of PSs and the light source (Figure 1B). A series (S) circuit-type power transmitting device and a parallel (P) circuit-type power receiving device were designed based on simulations, and the power receiving devices equipped with LEDs that emit blue light (470 nm) were prepared to irradiate the Ir complex **7** which, in our previous study [26], was reported to induce cancer cell death by photoirradiation at 465 nm and used as a PS in this study. The generation of ^1^O_2_ from ^3^O_2_ upon the photoirradiation of **7** in the WPT system was examined, and it was found that a larger voltage, a longer photoirradiation time, and a closer distance between the power transmission devices and the power receiving devices produce a larger dose of ^1^O_2_. Finally, we report on the induction of cell death in HeLa S3 cells (human cervical carcinoma cells) after the photoirradiation of **7** using the WPT-PDT system developed in this study.

## 2. Results and Discussions

### 2.1. Design and Preparation of Devices for Wireless Power-Transmission (WPT) System

**Figure 2:**
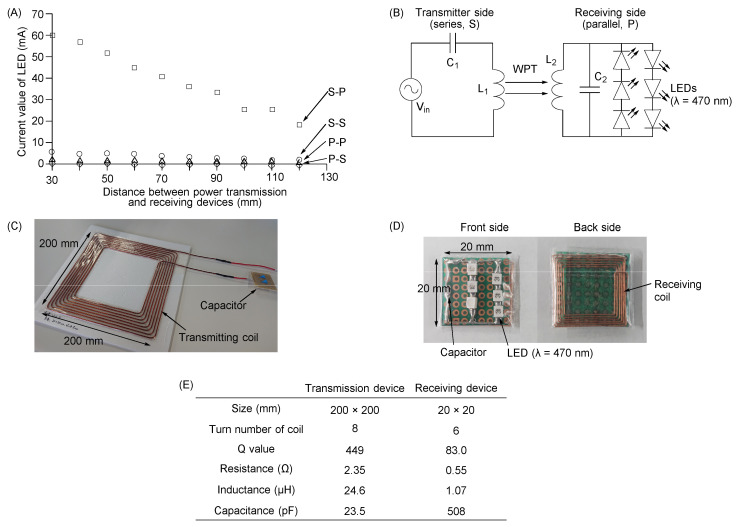
Design and preparation of the PT and PR for a WPT system. (**A**) The results of simulations of the relationship between the PT and PR (30–120 mm) using the electronic circuit simulation software “LTspice” and the current values of the LEDs on the receiving device for four basic circuits: S-S (open circles), S-P (open squares), P-S (open triangles), and P-P (open diamonds) (the circuit of the power-transmitter coil)-(the circuit of the power-receiving coil) (estimated input voltage was 10 V). (**B**) A schematic circuit (S-P) of the PT and PR; *V_in_*: an AC power supply, *C*_1_ and *C*_2_: capacitors, and *L*_1_ and *L*_2_: transmitter and receiver coils. (**C**) A picture of the transmitting device (200 mm × 200 mm). (**D**) A picture of the receiving device containing 6 LEDs (λ = 470 nm) (20 mm × 20 mm). (**E**) Representative parameters of the PT and PR.

Prior to the experiments, the relationship between the circuit of the power transmitter (PT) and the power receiver (PR) and the current values of LEDs for the design of the PT and PR was simulated using the electronic circuit simulation software “LTspice”. The circulated values of LED (mA) were estimated using an input voltage of 10 *V_rms_* (*rms*: root mean square, *V_rms_* = *V_peak_* × (1/√2)), as shown in Figure 2A. The input voltages used in this paper are root mean square (*RMS*) values because they are alternating current (AC) voltages. In these simulations, we tested four combinations of series (S) circuit-type and parallel (P) circuit-type PTs and PR (S-S, S-P, P-S, and P-P), in which capacitors (*C*_1_ and *C*_2_) and inductors (*L*_1_ and *L*_2_) are connected in series or in parallel with an input voltage of 10 V (alternating current), at different distances between the PT and PR (Figure S1 in the Supporting Information). As shown in Figure 2A, the current values for the LED in the S-P circuit ((the circuit of power transmitter coil)-(the circuit of power receiving coil)) were highest among these four circuit systems, and they decrease with increasing distance between the PT and PR (60 mA at 30 mm between the PT and PR). These results allowed us to design a combination of an S circuit type of the PT and a P circuit type of the PR for WPT through magnetic resonance coupling between the PT and PR as shown in Figure 2B, in which *V_in_* represents an electric power generator, *C*_1_ and *C*_2_ indicate capacitors, and *L*_1_ and *L*_2_ denote transmitter and receiver coils, respectively. Figure 2C,D display the pictures of the PT and the PR equipped with six LEDs [52], respectively, which emit blue light (λ = 470 nm), and their typical structural and electrical parameters are listed in Figure 2E.

### 2.2. Evaluation of a Power Transmitting Device and a Power Receiving Device in the WPT System

**Figure 3:**
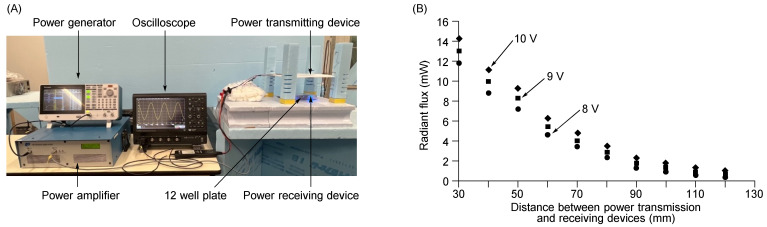
Evaluation of the function of the PT and PR in this study. (**A**) Experimental equipment consisting of an electric power generator, an AC power amplifier, an oscilloscope, and the PT and PR. (**B**) Measurement of radiant flux from LEDs on the PR at different input voltages (8 V (●), 9 V (■), and 10 V (♦)) with different distances between the PT and PR (30–120 mm).

The radiant flux (mW) from the LEDs on the PR was measured at different input voltages with different distances between the PT and PR. The operating frequency used in this study was 6.78 MHz, which is allowed in the use of radio frequency (RF) energy for industrial, scientific, and medical (ISM) purposes and is known as an ISM radio band. Figure 3A shows the experimental equipment consisting of an AC power generator, a power amplifier, an oscilloscope, and the PT that was used in this study. The magnetic field around the PT was generated by an input voltage (8~10 V) to transfer electric energy to the PR, in which six LEDs emit blue light (λ = 470 nm). As shown in Figure 3B, the radiant flux from the LEDs on the power receiving device is increased in a voltage-dependent manner (7.1 mW, 8.3 mW, and 9.2 mW at 8 V, 9 V, and 10 V, respectively, with a distance of 50 mm between the PT and PR) and decreased in a distance-dependent manner (9.2 mW, 3.4 mW, and 1.1 mW with a distance of 50 mm, 80 mm, and 110 mm, respectively, at 10 V), all of which indicate the relationship between the radiant flux from the LEDs, the input voltage, and the distance between the PT and PR.

### 2.3. Generation of Singlet Oxygen by the Wireless Power Transmission System

**Figure 4:**
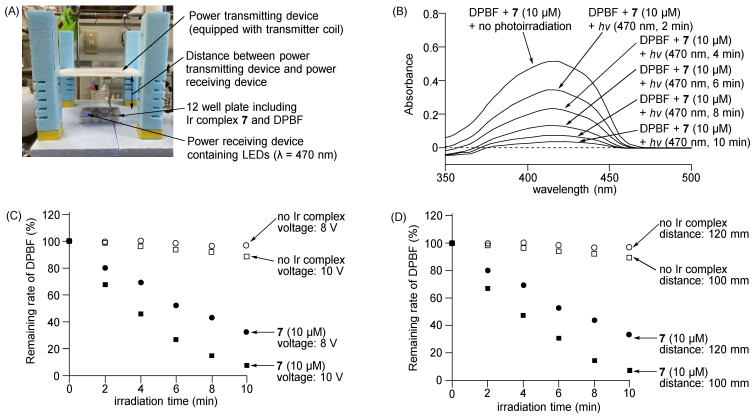
(**A**) Experimental equipment used for WPT. (**B**) UV spectra of DPBF (50 µM) upon the photoirradiation (470 nm) of **7** (10 µM) in DMSO/H_2_O (3/2) including HCl (50 µM) in the WPT system for 0–10 min at 10 V at a distance of 120 mm between the PT and PR. (**C**) Effect of voltage on the photooxidation of DPBF (50 µM); (◯) 8 V and (☐) 10 V in the absence of **7** (10 µM), (●) 8 V, and (■) 10 V in the presence of **7** (10 µM). The distance between the PT and PR was 120 mm. (**D**) Effect of distance between the PT and PR on the photooxidation of DPBF (50 µM); (◯) 120 mm and (☐) 100 mm in the absence of **7** (10 µM), (●) 120 mm and (■) 100 mm in the presence of 7 (10 µM) at 8 V.

It was previously confirmed that the photoirradiation of **7** at 465 nm induces the generation of singlet oxygen (^1^O_2_) from triplet oxygen (^3^O_2_) in aqueous solutions at acidic~basic pH [30]. The generation of ^1^O_2_ from ^3^O_2_ by the irradiation of Ir complex **7** at 470 nm in the WPT system (in a mixture of DMSO/H_2_O (3/2)) was evaluated using 1,3-diphenylisobenzofuran (DPBF), the absorbance of which at 415 nm is decreased upon reacting with ^1^O_2_ (Figure 4A) [25,26,53]. Figure 4B shows the changes in UV-Vis spectra of DPBF by the photoirradiation of **7** (10 µM), in which the absorbance of DPBF at 415 nm was decreased upon photoirradiation for 2–10 min, indicating the decomposition of DPBF by reacting with ^1^O_2_ generated from ^3^O_2_. The effect of voltage and distance between the PT and PR on the generation of ^1^O_2_ was also examined (Figure 4C,D). DPBF was decomposed by 68% and 93% after photoirradiation for 10 min at 8 V and 10 V, respectively, indicating a positive relationship between the voltage and ^1^O_2_ generation (Figure 4C). It was also found that a closer distance between the PT and PR resulted in a higher decomposition rate of DPBF (94% at a distance of 100 mm and 68% at a distance of 120 mm) as shown in Figure 4D. It should be noted that the decomposition of DPBF proceeds very slowly in the absence of **7** (open circles and squares in Figure 4C,D).

### 2.4. Induction of Cell Death in HeLa S3 Cells upon the WPT-Based Photoirradiation of Ir Complex **7**

**Figure 5:**
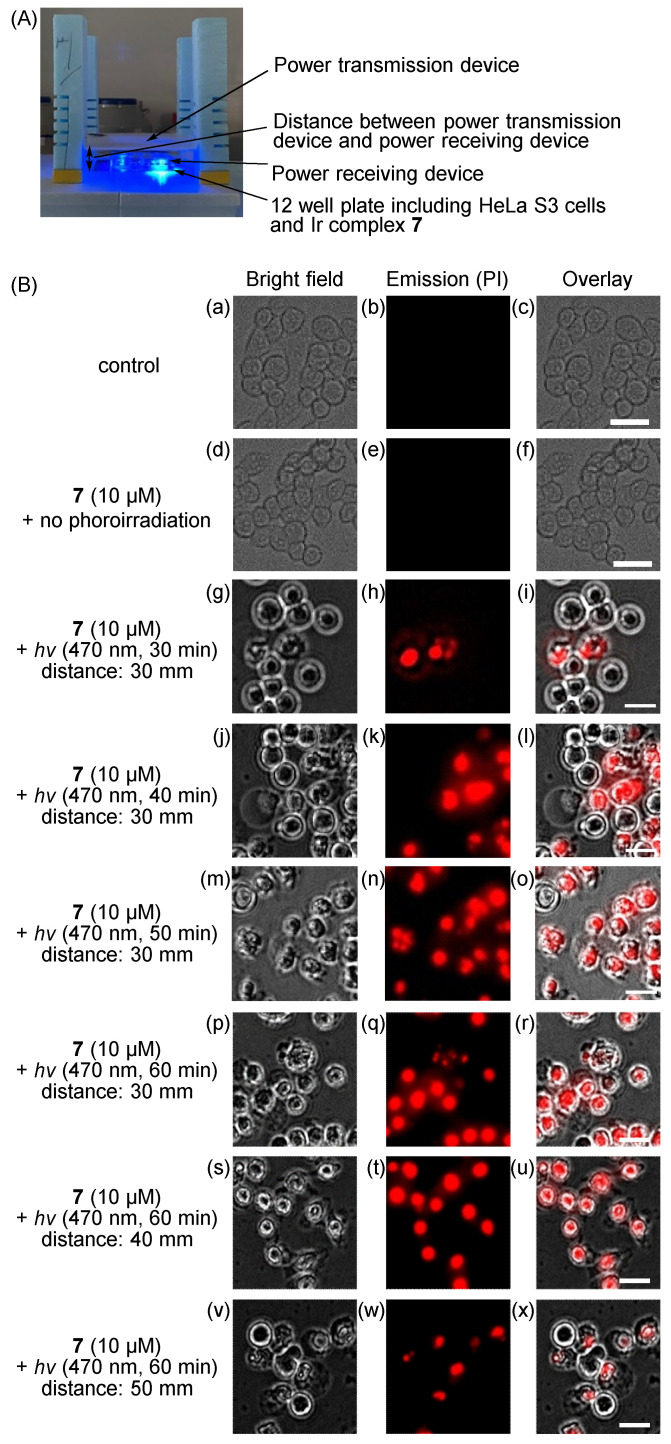
(**A**) A picture of the experimental devices for the WPT-based photoirradiation of HeLa S3 cells in this study. (**B**) Microscopic images of HeLa S3 cells (**a**–**c**) in control, (**d**–**f**) in the presence of **7** (10 µM) without photoirradiation, and (**g**–**x**) in the presence of **7** with photoirradiation at 470 nm for 30–60 min for different distances between the PT and PR at 10 V. (**a**,**d**,**g**,**j**,**m**,**p**,**s**,**v**) Bright field images of HeLa S3 cells, (**b**,**e**,**h**,**k**,**n**,**q**,**s**,**w**) emission images of PI (excitation at 540 nm and emission at 605 nm), (**c**) overlay image of (**a**,**b**), (**f**) overlay image of (**d**,**e**), (**i**) overlay image of (**g**,**h**), (**l**) overlay image of (**j**,**k**), (**o**) overlay image of (**m**,**n**), (**r**) overlay image of (**p**,**q**), (**u**) overlay image of (**s**,**t**), and (**x**) overlay image of (**v**,**w**). The scale bar (white) is 20 µm.

The induction of cell death in HeLa S3 cells by the photoirradiation of **7** using the WPT system was conducted (Figure 5A). Figure 5B, Figures S2 and S3 in the Supporting Information show luminescent microscopic images of HeLa S3 cells after photoirradiation at 470 nm in the absence or presence of **7** (10 µM). Dead cells were detected by propidium iodide (PI) dye, which exhibits a strong red emission by intercalating with DNA in dead cells. As shown in Figure 5B and Figure S2 in the Supporting Information, cell death was negligibly induced in the control samples (Figure 5B(a–c)), in the presence of **7** without photoirradiation (Figure 5B(d–f)), and with photoirradiation at 470 nm in the absence of **7**. The swelling of the cell membrane and strong red emission from PI were observed when **7** was excited at 470 nm for 30–60 min with a distance of 30–50 mm between the PT and PR devices (Figure 5B(g–x) and Figure S3 in the Supporting Information). These results indicate that necrosis in HeLa S3 cells is induced by ^1^O_2_ generated from ^3^O_2_ upon the photoirradiation of **7**, as reported previously [24,25,26].

**Figure 6:**
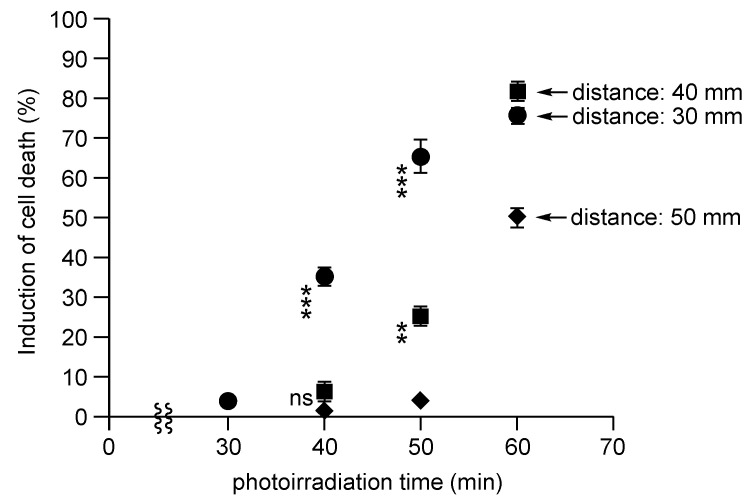
Induction of cell death in HeLa S3 cells monitored by PI staining after photoirradiation at 470 nm through WPT in the presence of **7** (10 µM) for different photoirradiation times (30–60 min) and at different distances between the PT and PR devices ((●) 30 mm, (■) 40 mm, and (♦) 50 mm). The ratio of dead cells = (number of cells stained with PI/total number of cells) × 100. The statistical analysis is performed at 40 min and 50 min, respectively. ** *p* < 0.01, *** *p* < 0.001, ns: not significant.

The effect of the photoirradiation time (irradiation at 470 nm) on cell death in HeLa S3 cells was examined. As shown in Figure 6, the efficiency of cell death in HeLa S3 cells increases with decreasing distance between the PT and PR (65%, 26%, and 4% for distances of 30, 40, and 50 mm, respectively, after the photoirradiation for 50 min) and with longer photoirradiation time (4%, 35%, 65%, and 75% for 30, 40, 50, and 60 min, respectively, with a PT-PR distance of 30 mm).

**Figure 7:**
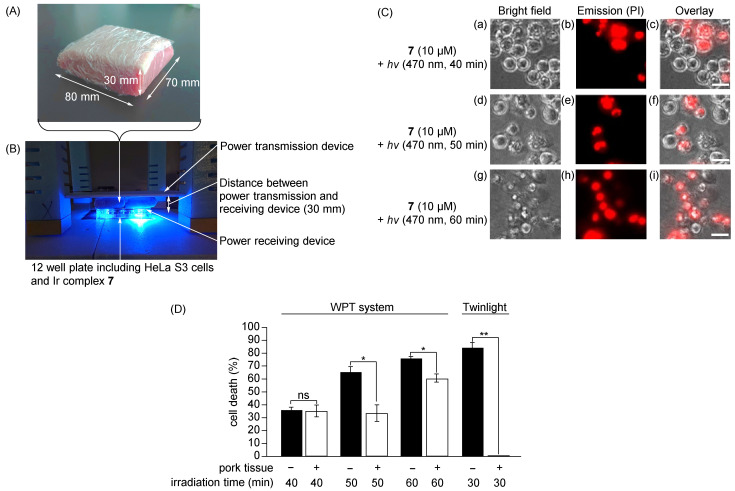
(**A**) A block of pork tissue (70 mm × 80 mm × 30 mm) was used as a model animal tissue. (**B**) Experimental devices for PDT of HeLa S3 cells by photoirradiation using WPT in the presence of a block of pork tissue between the PT and PR. (**C**) Microscopic images of HeLa S3 cells after the photoirradiation of 7 (10 µM) at 470 nm for (**a**–**c**) 40 min, (**d**–**f**) 50 min, and (**g**–**i**) 60 min with a distance of 30 mm between the PT and PR (**a**,**d**,**g**). Bright field images of HeLa S3 cells, (**b**,**e**,**h**) emission images of PI (excitation at 540 nm and emission at 605 nm), (**c**) overlay image of (**a**,**b**), (**f**) overlay image of (**d**,**e)**, (**i**) overlay image of (**g**,**h**). (**D**) Induction of the death of HeLa S3 cells in the absence (black bars) or presence (white bars) of a block of pork tissue monitored by PI staining upon the photoirradiation of 7 (10 µM) using the Twinlight (465 nm) for 30 min at a distance of 50 mm above the cells or WPT system (470 nm) for 40–60 min at a distance of 30 mm between the PT and PR. The ratio of dead cells = (number of cells stained with PI/total number of cells) × 100. The statistical analysis is performed between the cell death in the presence and absence of pork tissue. * *p* < 0.05, ** *p* < 0.01, ns: not significant.

Next, we examined the WPT-PDT against HeLa S3 cells in the presence of animal tissues between the PT and PR to mimic a situation, in which Ir(III) complexes (or other photosensitizers) are irradiated via the WPT system in the body. Namely, a block of pork (size: 70 mm × 80 mm (width and length) × 30 mm (thickness)) (Figure 7A) was inserted between the PT and PR as shown in Figure 7B. Figure 7C shows microscopic images of HeLa S3 cells after the photoirradiation of **7** at 470 nm for 40–60 min with a PT-PR distance of 30 mm. It should be mentioned that the radiant flux was measured to be 14.3 mW and 12.3 mW in the absence and presence of a block of pork tissue (when the distance between the PT and the PR was 30 mm at the input voltage of 10 V), respectively, suggesting that radiant flux is somewhat reduced (ca. 2 mW) by the presence of a block of pork tissue. The findings indicate that the blue emission from the LEDs (Figure 7B) induced cell death in HeLa S3 cells (PI-stained cells in Figure 7C), while the radiant flux is somewhat reduced by the insertion of pork tissue; therefore, longer photoirradiation time was required for the induction of cell death in the case of the inserted pork tissue compared to its absence (Figure 7D).

The photoinduced cell death of HeLa S3 cells in the presence of **7** was also examined by using Twinlight as a light source, in which electric power is transferred from the power generator to LEDs via a direct connection (Figure S4 in the Supporting Information). The induction of cell death in HeLa S3 cells was negligible in the absence of **7** (Figure S4B(a–c,g–i) in the Supporting Information). Approximately 84% of the HeLa S3 cells were killed after the direct photoirradiation of **7** at 465 nm for 30 min at a distance of 50 mm above the cells by using the Twinlight LED (wired LED device) (Figure 7D and Figure S4B(d–f) in the Supporting Information). Cell death was barely observed in the case where the pork sample was inserted between the cells and the Twinlight LEDs because only negligible levels of blue light from the Twinlight LEDs pass through the pork tissue (Figure 7D and Figure S4B(j–l) in the Supporting Information). These results indicate that the WPT-based PDT system used in this study could be applied to the treatment of deep-seated tumors that are difficult to irradiate by shorter wavelength light from outside the body.

## 3. Materials and Methods

### 3.1. General Information

All reagents and solvents purchased were the highest commercial quality and used without further purification otherwise noted. Dimethyl sulfoxide (DMSO) was purchased from KANTO chemicals. Propidium iodide (PI), benzylpenicillin potassium, and streptomycin sulfate were purchased from Fujifilm Wako Chemicals. 1,3-Diphenylisobenzofuran (DPBF) was purchased from Sigma-Aldrich. Minimum essential medium (MEM) and phosphate-buffered saline (PBS) were purchased from Nacalai Tesque. Fetal bovine serum (FBS) was purchased from Capricorn Products Inc. The Ir complex **7** was synthesized according to our previous paper [26], a stock solution of which in DMSO was prepared and stored at 0 °C prior to use. UV-Vis absorption spectra of DPBF were measured on a JASCO V-550 UV-vis spectrophotometer. The microscopic images of HeLa S3 cells were observed on fluorescent microscopy (Biorevo, BZ-x800, Keyence, Amagasaki, Japan).

### 3.2. Design and Evaluation of the Function of the Power Transmitting Device and Power Receiving Device for Wireless Power Transmission (WPT)

In designing the WPT system, LTspice (Analog Devices, Inc., Wilmington, MA, USA) was used as the electronic circuit simulation software. The following parameters were fixed when performing the simulation: drive frequency, input voltage, capacitance, and inductance (capacitance and inductance were measured using an impedance analyzer (E4900A, Keysight Technologies, Santa Rosa, USA). In this simulation, the variation in the transfer distance is considered a variation of the coupling between the transmitting and receiving coils, and the coupling factor is used as a variable parameter. S-S, S-P, P-S, and P-P denote the connection mode of capacitors and inductors in the equivalent circuit schematic, with the former part denoting the transmitting side and the latter part denoting the receiving side. Here, “S” denotes a series connection of elements, and “P” denotes a parallel connection of elements. Since circuit characteristics vary depending on how the elements are connected, they are used in different ways depending on the application.

WPT equipment comprises two major components: the transmitting side and the receiving side. The 6.78 MHz sine wave signal generated by the arbitrary function generator (AFG31021, Tektronix, Beaverton, USA) was amplified using a linear power amplifier (2100L, Electronics & Innovation, Rochester, USA) and the input to the transmitting device. Considering the withstand voltage of the compensating capacitor (10 pF × 2) on the transmitting side, the input voltage was set to 10 V_rms_ after amplification using a high-definition oscilloscope (HDO6034A, TELEDYNE LECROY, New York, USA). The receiving device comprised a six-turn spiral coil, two compensating capacitors, and six LEDs. Capacitors (120 pF capacitor (GRM1882C1H121JA01, Murata Manufacturing, Nagaokakyo, Japan), 390 pF capacitor (GRM2162C1H391JA01, Murata Manufacturing, Japan)) and LED (CLM1B-BKW-CTAUB363, CREE, Durham, USA) with 470 nm as typical dominant wavelength were placed in parallel on the top side of the thin glass epoxy circuit board (UB-THN01, Sunhayato, Tokyo, Japan). The spiral coil was placed on the bottom side and connected in parallel as well. All components were mounted using soldering materials for high-density integrated circuit boards (SD-60, TAIYO ELECTRIC INDUSTRY, Tokyo, Japan). To prevent mechanical and moisture damage and for insulation purposes, the receiving devices were encapsulated in a 0.08 mm thick polyethylene package using a sealer (AP-400). The radiant flux of the LEDs was measured using an optical power and energy meter (PM100D, Thorlabs, Newton, MA, USA).

### 3.3. Cell Culture

HeLa S3 cells were incubated in MEM that contained 10% heat-inactivated FBS and 1% penicillin/streptomycin at 37 °C in a humidified 5% CO_2_ incubator.

### 3.4. Measurement of Singlet Oxygen (^1^O_2_) by Using DPBF

DPBF (300 μM) in DMSO (0.5 mL) was added to a solution of the Ir complex **7** (12 μM) and HCl (60 μM) in DMSO/H_2_O (1.3/1.2 (*v*/*v*)) (2.5 mL). The resulting solutions containing **7** (10 μM), DPBF (50 μM), and HCl (50 μM) were photoirradiated at 470 nm for 0–10 min at room temperature in the dark room (as shown in Figure 5A) by the WPT system at a voltage of 8–10 V with the distance between the PT and PR of 100 mm and 120 mm, after which the changes in UV-Vis spectra of DPBF were measured.

### 3.5. Photoinduced Cell Death in HeLa S3 Cells by the WPT System

In a 12-well plate, HeLa S3 cells (5.0 × 10^5^ cells) in MEM (0.8 mL) were seeded and incubated overnight at 37 °C in a humidified 5% CO_2_ incubator, to which a solution of 7 (50 μM) in MEM (0.2 mL) was added. The resulting solutions containing 7 (10 μM) were incubated for 30 min at 37 °C under 5% CO_2_. After washing the cells with PBS, MEM (0.5 mL) was added, and the resulting solutions were irradiated at 470 nm for 30–60 min at room temperature in the WPT system at a voltage of 10 V with the distance between the PT and PR of 30–50 mm in the absence or presence of a block of pork tissue (these expeirments were carried out in the dark room, as shown in Figure 7B). For the wired (not wireless) photoirradiation, HeLa S3 cells were irradiated at 465 nm by using Twinlight (RELYON, Tokyo, Japan) for 30 min from a distance of 50 mm above the cells. The solutions were incubated for 24 h at 37 °C in a humidified 5% CO_2_ incubator, to which propidium iodide (PI) (1.5 mM) in PBS (10 μL) was added. After incubating for 30 min at 37 °C under an atmosphere of 5% CO_2_ followed by washing with PBS, the cells were observed by fluorescent microscopy (Biorevo, BZ-x800, Keyence). Excitation at 540 nm and emission at 605 nm were used for PI. The numbers of live cells and dead cells (stained with PI) were counted manually, and dead/live cell ratios were calculated with the following equation: ratio of dead cells = (number of cells stained with PI/total number of cells) × 100. Statistical significance was determined by T-test or Dunnett’s multiple comparison test using Graph Pad Prism 9.4.1. It should also be mentioned that several methods were used for the evaluation of photoinduced anticancer activity of our previous Ir(III) complexes [24,25,26] and it was confirmed that PI staining experiments give most reliable and reproducible results. The same situation was observed in this work, in which 12-well plates were used for the measurement of cell death. Therefore, PI staining experiments were used in this work.

## 4. Conclusions

In this manuscript, we report on a prototype of a wireless power-transmission-based photodynamic therapy (WPT-PDT) system that uses shorter wavelength light (470 nm) for the excitation of the Ir complex **7**. The series (S) circuit-type power transmitting device (PT) and the parallel (P) circuit-type power receiving device (PR) were designed and prepared based on the simulation of the relationship between the circuit of the PT and PR and the current values of LEDs. It was found that the radiant flux from the LEDs on the PR is increased in an input voltage-dependent manner and decreased in a distance-dependent manner between these two devices.

The generation of ^1^O_2_ was confirmed using DPBF, whose absorbance at 415 nm is decreased upon photoirradiation at 470 nm in the presence of **7**. The results of microscopic observations of HeLa S3 cells revealed that cell death is induced after the photoirradiation of **7** at 470 nm for 30–60 min through the WPT system with a distance of 30–50 mm between the PT and PR, and the ratio of dead cells is increased in an irradiation time- and distance-dependent manner. In addition, the induction of cell death was observed when a block of pork tissue (as a model of animal tissue) was inserted between the two devices, while cell death is negligibly induced when Twinlight was used as a light source from the upper side of the pork tissue. The WPT system developed in this study could be useful for WPT-PDT and WPT-PIT systems with any combinations of light sources and photosensitizers, such as analogs of porphyrins, phthalocyanines, and related compounds that can be excited by red-color or near-infrared (NIR) light that penetrates the skin for use in treating deep-seated tumors. In addition, we assume that our WPT-PDT systems have the potential for use in a battery-free endoscopy capsule and related instruments for the treatment of cancers, which are difficult to treat by implantable wireless WPT systems for the repeated PDT (metronomic PDT) [51]. Our next work, therefore, will be the design of a smaller WPT-LED that can fit into drug capsules, and so on.

## Data Availability

Not applicable.

## Supplementary Materials

The following supporting information can be downloaded at: https://www.mdpi.com/article/10.3390/molecules28031433/s1, Figure S1: Schematic presentation of the 4 combinations of series (S) circuit type and parallel (P) circuit type of the PT and PR. (a) S-S, (b) S-P, (c) P-S, and (d) P-P used in this study. Figure S2: Microscopic images of HeLa S3 cells after photoirradiation at 470 nm for 60 min in the WPT system in the absence of the Ir complex 7 (10 M) with distances of (a–c) 30 mm, (d–f) 40 mm, and (g–i) 50 mm between the PT and PR (see Figure 2 in the text). (a,d,g) Bright field images of HeLa S3 cells, (b,e,h) emission images of PI (excitation at 540 nm and emission at 605 nm for PI imaging), (c) overlay image of (a,b), (f) overlay image of (d,e), and (i) overlay image of (g,h). Scale bars (white) indicate 20 m. Figure S3: Microscopic images of HeLa S3 cells after photoirradiation at 470 nm for 40 and 50 min in the presence of Ir complex 7 (10 M) in the WPT system with photoirradiation at 470 nm for 40 and 50 min with distances of (a–f) 40 mm and (g–l) 50 mm between the PT and PR. (a,d,g,j) Bright field images of HeLa S3 cells, (b,e,h,k) emission images of PI (excitation at 540 nm and emission at 605 nm for PI imaging), (c) overlay image of (a,b), (f) overlay image of (d,e), (i) overlay image of (g,h), and (l) overlay image of (j,k). Scale bars (white) indicate 20 m. Figure S4: (A) The experimental equipment for PDT of HeLa S3 cells by photoirradiation at 465 nm using a Twinlight, which consists of wired (not wireless) LED. (B) Microscopic images of HeLa S3 cells after photoirradiation at 465 nm for 30 min in the presence of Ir complex 7 (10 M) by Twinlight in the absence (a–f) or presence (g–l) of pork tissue. (a,d,g,j) Bright field images of HeLa S3 cells, (b,e,h,k) emission images of PI (excitation at 540 nm and emission at 605 nm for PI imaging), (c) overlay image of (a,b), (f) overlay image of (d,e), (i) overlay image of (g,h), and (l) overlay image of (j,k). Scale bars (white) indicate 10 m.

## Sample Availability

Samples of the compounds are available from the authors.
