# Supplementary material for: Development of Wireless Power-Transmission-Based Photodynamic Therapy for the Induction of Cell Death in Cancer Cells by Cyclometalated Iridium(III) Complexes
Source: Molecules. 2023 Feb 2;28(3):1433. doi: 10.3390/molecules28031433 (PMC9919167; doi:10.3390/molecules28031433)
Supplement: Supplementary file 1 [file molecules-28-01433-s001.zip › molecules-2146854-supplementary.pdf]

Development of Wireless Power-Transmission-Based Photodynamic Therapy for the Induction of Cell Death in Cancer Cells by Cyclometalated Iridium(III) Complexes

Kenta Yokoi [1], Yoshitaka Yasuda [2], Azusa Kanbe [1], Takehiro Imura [2,*] and Shin Aoki [1,3,4,*]

[1] Faculty of Pharmaceutical Sciences, Tokyo University of Science, 2641 Yamazaki, Noda 278-8510, Chiba, Japan; yokoken4513@gmail.com (K.Y.); 3b21519@ed.tus.ac.jp (A.K.)
[2] Faculty of Science and Technology, Tokyo University of Science, 2641 Yamazaki, Noda 278-8510, Chiba, Japan; 7321588@ed.tus.ac.jp
[3] Research Institute for Science and Technology (RIST), Tokyo University of Science, 2641 Yamazaki, Noda 278-8510, Chiba, Japan
[4] Research Institute for Biomedical Sciences (RIBS), Tokyo University of Science, 2641 Yamazaki, Noda 278-8510, Chiba, Japan
* Correspondence: imura.takehiro@rs.tus.ac.jp (T.I.); shinaoki@rs.tus.ac.jp (S.A.); Tel.: +81-4-7121-3670 (S.A.)

Contents

Figure S1. Schematic presentation of the 4 combinations of series (S) circuit type and parallel (P) circuit type of the PT and PR. (a) S-S, (b) S-P, (c) P-S, and (d) P-P used in this study.

Figure S2. Microscopic images of HeLa S3 cells after photoirradiation at 470 nm for 60 min in the WPT system in the absence of the Ir complex **7** (10 μM) with distances of (a-c) 30 mm, (d-f) 40 mm, and (g-i) 50 mm between the PT and PR (see Figure 2 in the text). (a, d, and g) Bright field images of HeLa S3 cells, (b, e, and h) emission images of PI (excitation at 540 nm and emission at 605 nm for PI imaging), (c) overlay image of (a) and (b), (f) overlay image of (d) and (e), and (i) overlay image of (g) and (h). Scale bars (white) indicate 20 μm.

Figure S3. Microscopic images of HeLa S3 cells after photoirradiation at 470 nm for 40 and 50 min in the presence of Ir complex **7** (10 μM) in the WPT system with photoirradiation at 470 nm for 40 and 50 min with distances of (a-f) 40 mm and (g-l) 50 mm between the PT and PR. (a, d, g, and j) Bright field images of HeLa S3 cells, (b, e, h, and k) emission images of PI (excitation at 540 nm and emission at 605 nm for PI imaging), (c) overlay image of (a) and (b), (f) overlay image of (d) and (e), (i) overlay image of (g) and (h), and (l) overlay image of (j) and (k). Scale bars (white) indicate 20 μm.

Figure S4. (A) The experimental equipment for PDT of HeLa S3 cells by photoirradiation at 465 nm using a Twinlight, which consists of wired (not wireless) LED. (B) Microscopic images of HeLa S3 cells after photoirradiation at 465 nm for 30 min in the presence of Ir complex **7** (10 μM) by Twinlight in the absence (a-f) or presence (g-l) of pork tissue. (a, d, g, and j) Bright field images of HeLa S3 cells, (b, e, h,

and k) emission images of PI (excitation at 540 nm and emission at 605 nm for PI imaging), (c) overlay image of (a) and (b), (f) overlay image of (d) and (e), (i) overlay image of (g) and (h), and (l) overlay image of (j) and (k). Scale bars (white) indicate 10 μm.

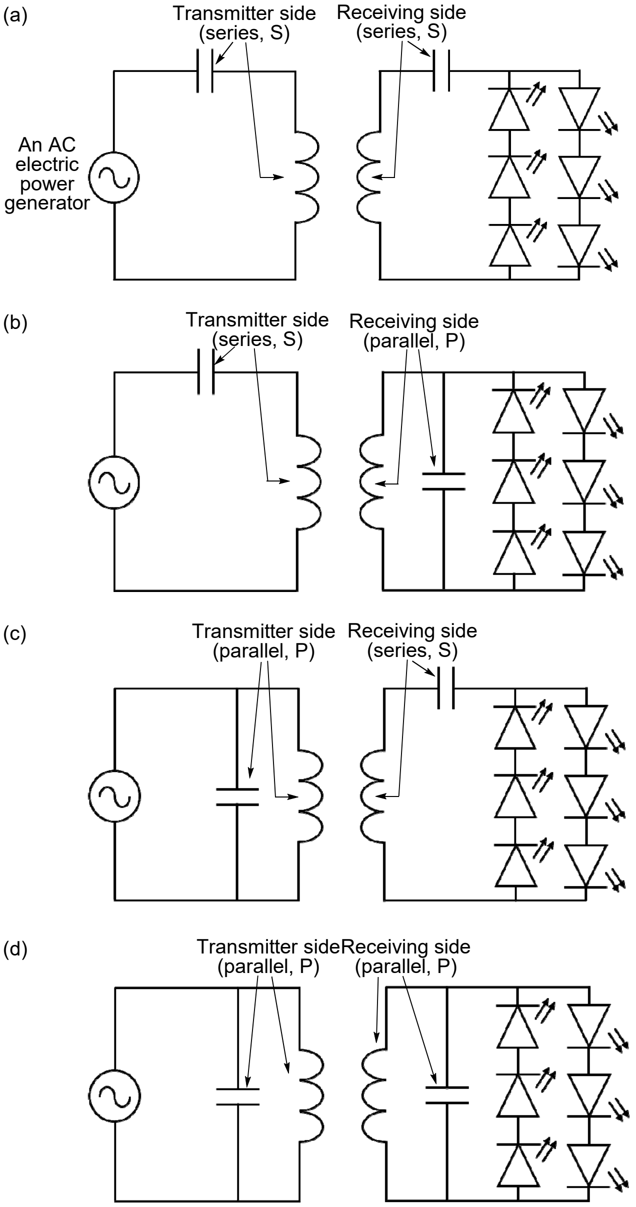

Figure S1. Schematic presentation of 4 combinations of series (S) circuit type and parallel (P) circuit type of the PT and PR. (a) S-S, (b) S-P, (c) P-S, and (d) P-P.

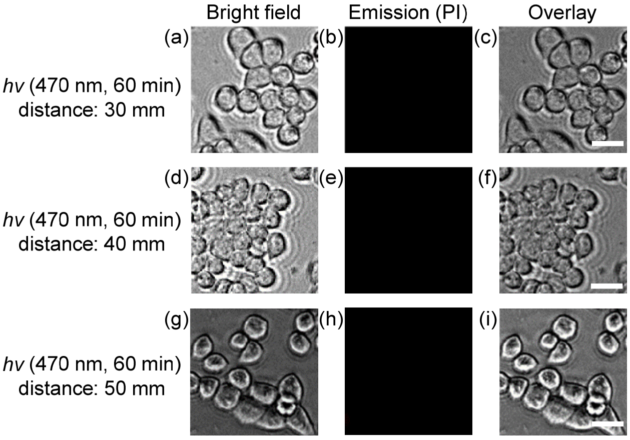

Figure S2. Microscopic images of HeLa S3 cells after photoirradiation at 470 nm for 60 min in the WPT system in the absence of the Ir complex **7** (10 μM) with distances of (a-c) 30 mm, (d-f) 40 mm, and (g-i) 50 mm between the PT and PR (see Figure 2 in the text). (a, d, and g) Bright field images of HeLa S3 cells, (b, e, and h) emission images of PI (excitation at 540 nm and emission at 605 nm for PI imaging), (c) overlay image of (a) and (b), (f) overlay image of (d) and (e), and (i) overlay image of (g) and (h). Scale bars (white) indicate 20 μm.

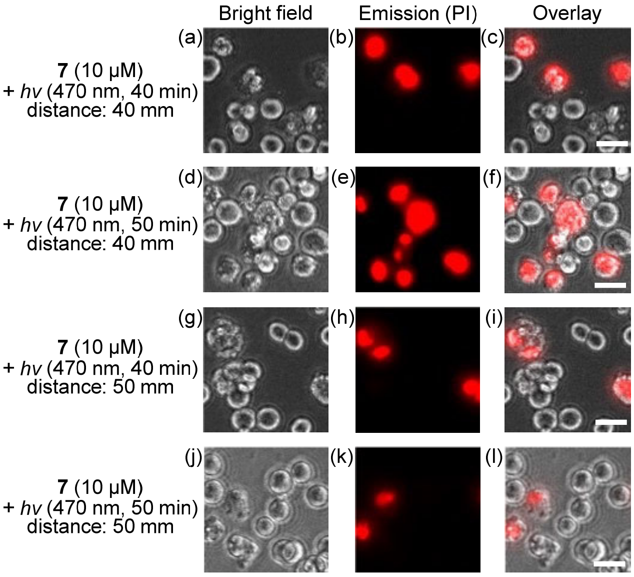

Figure S3. Microscopic images of HeLa S3 cells after photoirradiation at 470 nm for 40 and 50 min in the presence of Ir complex **7** (10 μM) in the WPT system with photoirradiation at 470 nm for 40 and 50 min with distances of (a-f) 40 mm and (g-l) 50 mm between the PT and PR. (a, d, g, and j) Bright field images of HeLa S3 cells, (b, e, h, and k) emission images of PI (excitation at 540 nm and emission at 605 nm for PI imaging), (c) overlay image of (a) and (b), (f) overlay image of (d) and (e), (i) overlay image of (g) and (h), and (l) overlay image of (j) and (k). Scale bars (white) indicate 20 μm.

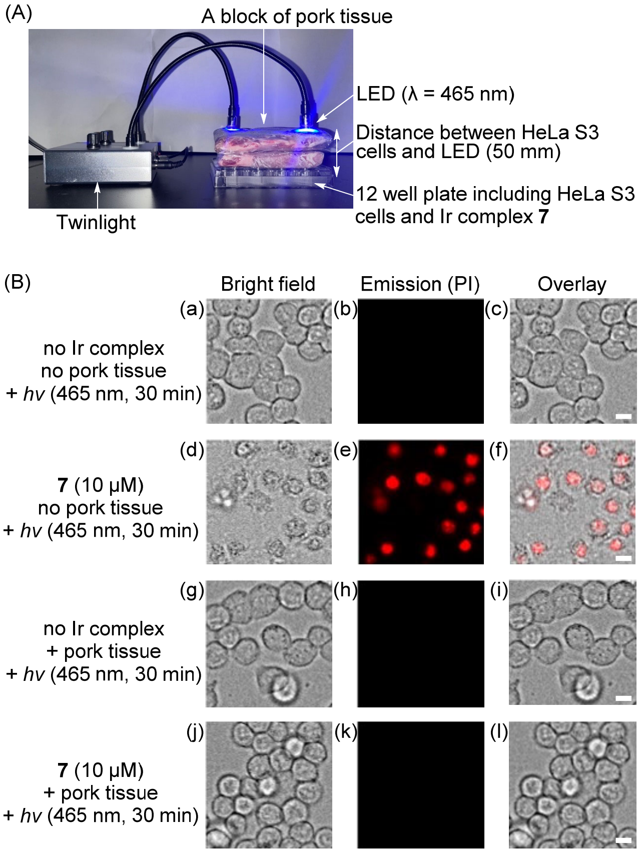

Figure S4. (A) The experimental equipment for PDT of HeLa S3 cells by photoirradiation at 465 nm using a Twinlight, which consists of wired (not wireless) LED. (B) Microscopic images of HeLa S3 cells after photoirradiation at 465 nm for 30 min in the presence of Ir complex **7** (10 μM) by Twinlight in the absence (a-f) or presence (g-l) of pork tissue. (a, d, g, and j) Bright field images of HeLa S3 cells, (b, e, h, and k) emission images of PI (excitation at 540 nm and emission at 605 nm for PI imaging), (c) overlay image of (a) and (b), (f) overlay image of (d) and (e), (i) overlay image of (g) and (h), and (l) overlay image of (j) and (k). Scale bars (white) indicate 10 μm.
